# Capsaicin-Cyclodextrin Complex Enhances Mepivacaine Targeting and Improves Local Anesthesia in Inflamed Tissues

**DOI:** 10.3390/ijms21165741

**Published:** 2020-08-10

**Authors:** Verônica Muniz Couto, Laura de Oliveira-Nascimento, Luiz Fernando Cabeça, Danilo Costa Geraldes, Juliana Souza Ribeiro Costa, Karin A. Riske, Michelle Franz-Montan, Fabiano Yokaychiya, Margareth K. K. Dias Franco, Eneida de Paula

**Affiliations:** 1 Department of Biochemistry and Tissue Biology, Institute of Biology, University of Campinas, Campinas 13083-862, SP, Brazil; veronica_couto85@yahoo.com.br (V.M.C.); depaula@unicamp.br (E.d.P.); 2 Faculty of Pharmaceutical Sciences, University of Campinas, Campinas 13083-871, SP, Brazil; danilo.geraldes8@gmail.com (D.C.G.); julianasrcosta@gmail.com (J.S.R.C.); 3 Technologic Federal University of Parana, Londrina 80060-000, PR, Brazil; luiscabeca@utfpr.edu.br; 4 Department of Biophysics, Federal University of Sao Paulo, Sao Paulo 04023-062, SP, Brazil; kariske@unifesp.br; 5 Department of Physiological Sciences, Piracicaba Dental School, University of Campinas, Piracicaba 13414-903, SP, Brazil; franzmontan@hotmail.com; 6 Nuclear and Energy Research Institute, IPEN–CNEN/SP, Sao Paulo 05508-000, SP, Brazil; fabiano.yokaichiya@gmail.com (F.Y.); margareth_franco@yahoo.com.br (M.K.K.D.F.); 7 Department of Quantum Phenomena in Novel Materials, Helmholtz-Zentrum, 14109 Berlin, Germany

**Keywords:** inflammation, mepivacaine, capsaicin, anesthesia, cyclodextrin

## Abstract

Acidic environments, such as in inflamed tissues, favor the charged form of local anesthetics (LA). Hence, these drugs show less cell permeation and diminished potency. Since the analgesic capsaicin (CAP) triggers opening of the TRPV1 receptor pore, its combination with LAs could result in better uptake and improved anesthesia. We tested the above hypothesis and report here for the first time the analgesia effect of a two-drug combination (LA and CAP) on an inflamed tissue. First, CAP solubility increased up to 20 times with hydroxypropyl-beta-cyclodextrin (HP-β-CD), as shown by the phase solubility study. The resulting complex (HP-β-CD-CAP) showed 1:1 stoichiometry and high association constant, according to phase-solubility diagrams and isothermal titration calorimetry data. The inclusion complex formation was also confirmed and characterized by differential scanning calorimetry (DSC), X-ray diffraction, and ^1^H-NMR. The freeze-dried complex showed physicochemical stability for at least 12 months. To test in vivo performance, we used a pain model based on mouse paw edema. Results showed that 2% mepivacaine injection failed to anesthetize mice inflamed paw, but its combination with complexed CAP resulted in pain control up to 45 min. These promising results encourages deeper research of CAP as an adjuvant for anesthesia in inflamed tissues and cyclodextrin as a solubilizing agent for targeting molecules in drug delivery.

## 1. Introduction

The presence of inflammation decreases the efficiency of local anesthetic (LA) agents in clinical procedures [1]. Since inflammation demands a higher amount of LA, the patient stands at risk of systemic toxicity, especially to the nervous and cardiovascular systems [2]. Therefore, new strategies that provide local anesthesia under inflammation are highly desirable. The reduced efficacy of LA under inflammation has been explained by a variety of hypothetical mechanisms, such as peripheral vasodilatation (increment in LA clearance), increased excitability of nerves, and pH reduction in the inflamed tissue [3,4]. Although no single mechanism could fully explain the failure of LA in such conditions, the reduced pH in the tissue is the most accepted one [5]. According to the Henderson–Hasselbalch equation [6], LA (pKa between 7.6–9.0) are mainly charged under acidic pH. Since the charged LA species poorly permeate the neuron cells, a necessary step to further bind the block site in the voltage-dependent sodium channel, low pH could provoke LA failure [7,8].

Capsaicin (CAP) is the active ingredient in *Capsicum* peppers, with major pharmacological application as analgesic [9,10]. Its mechanism of action includes binding to type 1 transient potential vanilloid receptor (TRVP1), a transmembrane channel for cations. This binding keeps the receptor in the open state, which allows crossing of charged molecules as large as lidocaine N-ethyl bromide [11] through it. Hence, our hypothesis is that the association of CAP with local anesthetics could effectively anesthetize inflamed tissues by LA intracellular delivery through TRPV1 pores. To test the assumption, mepivacaine was chosen as the local anesthetic. Mepivacaine has low systemic toxicity and offers intermediate duration of anesthesia with a rapid onset, characteristics that favor pain evaluation with in vivo models [12].

CAP formulation, as other hydrophobic molecules, requires the use of surfactants, organic solvents or complexation with other molecules for its aqueous solubilization [13]. Regarding complexation, cyclodextrins (CD) can dismiss the use of organic solvents without the drawbacks of surfactants in parenteral formulations [14,15]. These cyclic oligosaccharides form molecular inclusion complexes in their inner (nonpolar) cavity by non-covalent interactions, besides outer ring binding [14,16,17]. They also stand out in the market, with over 35 different pharmaceutical products marketed as CD complexes around the world [18]. Among the CDs, hydroxypropyl-beta-cyclodextrin (HP-β-CD) is approved for parenteral drug administration by the FDA. Therefore, we proposed a freeze-dried CAP:HP-β-CD complex to be further reconstituted with a LA solution, such as mepivacaine. To the best of our knowledge, this work constitutes the first assessment of anesthesia in inflamed tissues with the association of a local anesthetic and CAP.

## 2. Material and Methods

### 2.1. Material—Reagents, Drugs, and Excipients

Capsaicin (C_18_H_27_NO_3_, 95% purity, 305.4 molecular weight) was obtained from Cayman Chemical Co. (Cayman Chemical Co., Ann Arbor, MI, USA); mepivacaine hydrochloride (C_15_H_22_N_2_O, 99% purity, 246.35 molecular weight) was donated by Cristália. (Cristália *Produtos Químicos Farmacêuticos* Ltda, Itapira, São Paulo, Brazil); hydroxypropyl-β-cyclodextrin (degree of substitution 5, 1460 molecular weight, 100% purity), acetonitrile (HPLC grade), and sodium acetate were purchased from Sigma-Aldrich (Sigma-Aldrich Corporation, St. Louis, Missouri, USA); phosphoric acid was from Ecibra, Ecibra, São Paulo, São Paulo, Brazil); monobasic and dibasic potassium phosphate from Labsynth (Labsynth, São Paulo, São Paulo, Brazil); acetic acid from Merk (Merck, Kenilworth, New Jersey, USA). Ultrapure water was obtained with the Milli-Q system (Merck KGaA, Darmstadt, Germany).

### 2.2. Phase Solubility Study

The effects of HP-β-CD on the solubility of CAP was investigated according to the phase solubility method described by Higuchi and Connors [19]. In this experiment increasing concentrations of CD (0–10 mM) were added to a saturated CAP solution. The solutions were left under magnetic stirring for 48 h (350 rpm and 25 °C). Aliquots of each vial were centrifuged for 10 min (4100× *g*) and the supernatant was filtered through 0.45 μm (Millipore, Burlington, Massachusetts, USA) and diluted to determine CAP concentration by UV absorption at 280 nm. The phase solubility diagram was obtained by plotting the solubility of CAP, versus the concentrations of HP-β-CD (M^−1^). The apparent stability constant (Ks) were calculated by Equation (1), where S_0_ is the aqueous molar solubility of the drug, taken from the Y intercept of the plot:

(1) Ks=slopeS0(1−slope)

The complexation efficiency (CE) determines the adequate ratio of CDs to ensure maximum solubility of the complexed drug [20]. For the CAP-HP-β-CD complex, CE was calculated from the phase solubility plots, according to Equation (2). The CE values allow drug/CD ratio in the formulation, according to Equation (3) [20]:

(2) CE=slope(1−slope)

(3) $$Drug:CD=\frac{1/\left(CE+1\right)}{CE}$$

### 2.3. Isothermal Titration Calorimetry

A microcalorimeter MicroCal VP-ITC (Malvern, Malvern, United Kingdom) was used to assess the stoichiometry of complexation between HP-β-CD and CAP. The calorimeter cell was filled with CAP solution (0.4 mM) and the syringe was loaded with HP-β-CD solution (10 mM), both using 10% (V/V) ethanol as solvent. Cyclodextrin aliquots of 10 µL were consecutively injected into CAP solution at 25° C. Raw data was normalized and corrected for the heat of the dilution (titration of HP-β-CD solution in the 10% ethanol solution). The binding stoichiometry was determined by fitting the final data to a one-site interaction model using the Origin software version 8 (OriginLab, Northampton, Massachusetts, USA).

### 2.4. Collapse Temperature 

The collapse temperature was determined to design the freeze-drying cycle for the HP-β-CD-CAP complex (4:1 molar ratio). The test was performed in an Elipse E600 polarized light microscope (Nikon, Melville, New York, Japan) coupled to a freeze-drying module (Lyostat 2, model FDCS 196, Linkam Instruments) (Biopharma, Winchester, UK). The equipment was calibrated with aqueous NaCl solution (eutectic temperature = −21.1 °C). The process of freezing was carried out with ramps of 10 °C/min up to −60 °C, at 750.0 Torr. Then, the heating was performed in a ramp of 5 °C/min, up to 0 °C, under 0.1 Torr pressure. The collapse temperature was determined visually, corresponding to the temperature at which the total loss of the structure (between the dry product matrix and the sublimation interface) occurs [21].

### 2.5. Preparation of Freeze-Dried HP-β-CD-CAP 

HP-β-CD (1.00 g) and CAP (0.05 g) were diluted in 100 mL of ultrapure water, at 25 °C. The obtained solution (HP- β-CD-CAP, 4:1 molar ratio) was magnetic stirred (350 rpm) for 24 h and filtered through 0.45 µm filter (Millipore, Burlington, Massachusetts, USA). Then, 4 mL samples were transferred to each lyophilization vial. The parameters of freeze-drying were calculated with the Smart Freeze Dryer Technology software, with the sample inputs: collapse temperature, morphology, fill volume, and vial diameter.

The freeze-drying process was carried out in a Lyostar 3 (SP Scientific) pilot freeze-dryer, according to the cycle suggested by the SMART software: sample loading at 25 °C, cooling up to −40 °C at 1 °C/min; hold at −40 °C for 120 min; primary drying up to −15 °C at 0.5 °C/min and pressure of 150 mTorr; hold for step completion at −15 °C and 150 mTorr for 90 min; secondary drying up to 40 °C at 0.1 °C/min and 150 mTorr; hold for step completion at 40 °C for 360 min.

### 2.6. Scanning Electron Microscopy 

Scanning electron microscopy (SEM) was used to analyze the morphology of the complex. Solid samples of pure CAP, HP-β-CD, their physical mixture, and inclusion complex (freeze-dried) were glued to an aluminum stub using double-sided carbon tapes. The stubs were covered in gold under vacuum for 200 s (Sputter Coater SCD-050) to become electrically conductive. The samples were analyzed by secondary electron emission JSM 5800LV Scanning Electron Microscope (JEOL, Peabody, Massachusetts, USA).

### 2.7. Differential Scanning Calorimetry

Differential scanning calorimetry (DSC) was used to characterize CD complexes by comparing the thermal behavior of pure compounds, their physical mixtures, and inclusion complexes [22]. The samples (5 mg) were analyzed in an aluminum pan (TA instruments, New Castle, Delaware, USA) of 40 µL using a Differential Scanning Calorimeter DSC1. (Mettler Toledo, Columbus, Ohio, USA). The samples were heated from 20 to 200 °C at a heating rate of 5 °C/min. Dynamic N_2_ atmosphere (flow rate: 50 mL/min) was used as purge gas. An empty aluminum pan was used as reference.

### 2.8. Stability

Each vial of HP-β-CD-CAP was resuspended in 4 mL of ultrapure water, further evaluated in a long-term condition (25 ± 2 °C storage condition) with trimestral analysis for a 12-month period. Stability testing of new drug substances and products states that no “significant change” for a drug product was defined as a 5% change in assay (UV-Vis spectroscopy) from its initial value. The samples should also meet the acceptance criteria for appearance of the cake, pH value, and time for resuspension. Additionally, three replicates of lyophilized HP-β-CD-CAP were analyzed at the end of the storage period (12 months) for moisture content using a volumetric Karl Fischer, Titrator DL31 Mettler Toledo (Columbus, USA).

### 2.9. Powder X-ray Diffraction 

Powder X-ray diffraction (PXRD) was used to investigate the crystallinity degree or amorphization of the inclusion complex in the solid state. The diffractogram of pure compounds, their physical mixtures, and inclusion complexes were investigated in a benchtop X-ray diffractometer Miniflex II (Rigaku, Tokio, Japan) operating in the Bragg–Brentano reflection mode, equipped with a cooper tube, line Kα radiation X-ray source. The angular intervals used were 5° to 40°, at a rate of 5°/min in 2θ° angle, and step sizes of 0.05.

### 2.10. Nuclear Magnetic Resonance

NMR analyses were performed in a Bruker Avance III (Bruker Corporation, Billerica, Massachusetts, USA) operating at 400 MHz for ^1^H frequency. The samples were prepared in H_2_D solvent and transferred to a 5 mm tube for direct and indirect detection. The deuterium signal of the solvent was used as field lock and adjustment of the homogeneity of the magnetic field. Nuclear magnetic resonance spectrometry (NMR) can provide evidence of the complexation through changes in ^1^H chemical shift of free (CD or drug) molecules and the complex HP-β-CD-CAP. However, the use of specific sequences (Rotating Frame Overhauser Enhancement Spectroscopy (ROESY) and Diffusion Ordered Spectroscopy (DOSY)) should be used to prove complex formation000000is a homonuclear Nuclear Overhauser Effect (NOE) measurement and can determine an intramolecular and intermolecular interaction, providing information regarding spatial proximities between hydrogens. 2D ROESY spectra were acquired under spin-locked conditions and a mixing time of 300 ms. The spectra were calibrated at 4.70 ppm (H_2_O).

DOSY is an experiment that separates the signals of a mixture of components according to the size and shape of the molecules [23,24]. DOSY experiments were conducted with the pulse sequence ledbpgp2s, with a time diffusion of 0.06 s. The gradient pulse amplitudes had a decrease in resonance intensity of approximately 90–95% for the higher intensity gradients. The average acquisition time of the experiment was 40 min. The processing program (Dosy Toolbox) was run with data transformed using fn = 32 K. The diffusion coefficient and standard deviation of each species was given by the arithmetic mean of all coefficients of the same species. DOSY allows determination of the association constant (Ka), between guest molecules and CDs. Ka values are inferred from the complexed fraction (F_complexed_) of the guest molecule, and both values were calculated accordingly to Equations (4) and (5), where D_drug_ is the diffusion coefficient of the free drug, D_complex_ is the diffusion coefficient of the complex, D_cyclodextrin_ is the diffusion coefficient of free CD, and R is the molar concentration of non-complexed CD [25]:

(4) $$F_{\mathrm{complexed}}=\frac{\left[D_{\mathrm{drug}}-D_{\mathrm{complex}}\right]}{\left[D_{\mathrm{drug}}-D_{\mathrm{ciclodextrin}}\right]}$$

(5) $$\mathrm{Ka}=\frac{\left[D_{\mathrm{drug}}-D_{\mathrm{complex}}\right]}{\left[D_{\mathrm{drug}}-D_{\mathrm{ciclodextrin}}\right]\;\times \;R}$$

### 2.11. In Vitro Release Experiments

A “Franz-type” vertical diffusion cell system was used to evaluate the in vitro release of CAP, comparing the free (dissolved in 20% ethanolic solution) and complexed drug with HP-β-CD [26]. The diffusion system consists of two compartments: the donor one—containing 1 mL of the sample—and the acceptor compartment, containing 12 mL of buffer (10 mM phosphate buffered saline, pH 7.4) kept at 37 °C, under mild stirring to ensure sink condition [27]. A cellulose membrane (Spectrapore, Los Angeles, 1000 Da molecular exclusion pore) separated the two compartments. Aliquots (200 μL) were withdrawn from the acceptor compartment at fixed intervals (15, 30, 60, 45, 60, and 90 min, and every hour for 6 h) and the volume was replaced with fresh medium. CAP was quantified by HPLC, in a Waters Breeze 2 System (Waters, Milford, Massachussets, USA) at 280 nm and 30 °C, using a Luna^®^ C18 (2) reverse phase (25 × 4.6 mm, 5 μm) (Phenomenex, Torrance, California, USA), 30-µL injection volume, phosphoric acid (1:1000 v/v) and acetonitrile (7:3 v:v), at pH 2.4, and 1.0 mL/min flow rate.

### 2.12. Evaluation of Analgesia in Inflamed Tissue

The antinociceptive effect of the association between mepivacaine and complexed CAP (HP-β-CD-CAP) was evaluated in vivo. A sample of freeze-dried HP-β-CD-CAP was resuspended in 4 mL of mepivacaine hydrochloride solution (MVC) to final concentrations of 1.6 mM (0.05%) CAP and 2% MVC. MVC solution was previously sterilized by filtration 0.22 μm. (Millipore, Burlington, Massachusetts, USA). Male Swiss mice (30–35 g) were obtained from Centro de Bioterismo, University of Campinas, UNICAMP (CEMIB-UNICAMP, Campinas, São Paulo, Brazil) and housed in standard cages under a 12/12 h of light/dark cycle. All experiments were approved by the institutional animal care and use committee of Unicamp, meaning it follows the Brazilian national guideline, which is in line with the National Institutes of Health guide for the care and use of Laboratory animals (protocol # 4402-1).

The mechanical allodynia was analyzed in mice hind paw by a Dynamic Plantar Aesthesiometer (model 37450, Ugo Basile, Italy). The animals were placed in an elevated cage for 2 h before the test, for setting and training. After that, 25 μL of carrageenan (2%) was applied in the subplantar region of the right mice paw to induce inflammation [28]. After 4 h of injection, the basal measurement was performed with a thin steel rod (0–5 g for 20 s, 0.5 g/s). The anesthetic formulations were then applied into the same subplantar region of the right paw of the mice, and the pain threshold was evaluated every 15 min. One group of animals was injected with sterile saline, as the control group. After the mechanical stimulus, the time elapsed until paw withdrawal as well as the applied pressure value were automatically recorded. The measured times were relativized to the baseline time (latency/baseline × 100) and displayed accordingly to the duration of the test (min) [29].

## 3. Results and Discussion

### 3.1. Phase Solubility Profile

**Figure 1:**
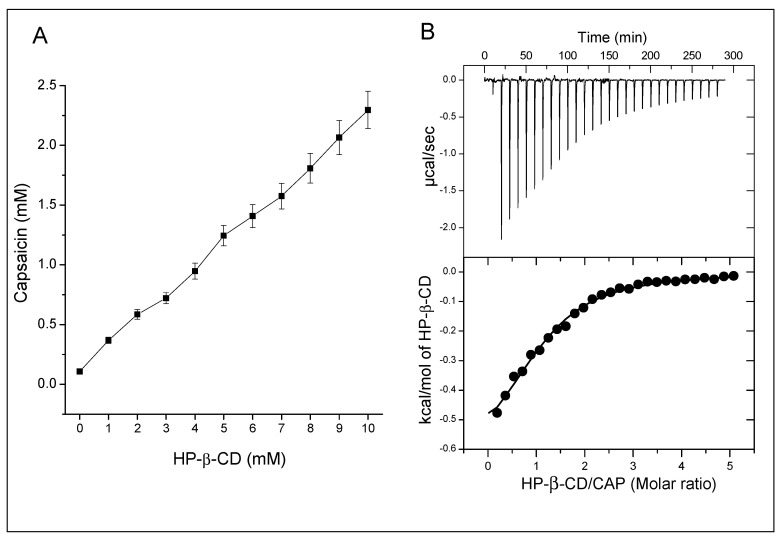
(**A**) Capsaicin solubility diagram, at different concentrations (0–10 mM) of hydroxypropyl-beta-cyclodextrin (HP-β-CD), at 25 °C. The results are expressed as mean ± SD (*n* = 3). (**B**) Isothermal titration calorimetric results obtained from titration of capsaicin (CAP) (0.4 mM) with HP-β-CD (10 mM in the syringe) at 25 °C. Upper panel, raw data of heat flow against time. Lower panel, heat per mol of injectant as a function of the heat of dilution. The line shows the one-site binding model.

The effect of HP-β-CD on CAP solubility was evaluated by addition of increasing concentrations of CD to a saturated solution of CAP. The phase solubility diagram showed an A-type phase-solubility profile (A_L_), with a first order linear increase (r^2^ = 0.996) in solubility as a function of the CD concentration (Figure 1A) [19]. The apparent solubility of CAP improved up to 20 times (2.2 mM of CAP with 10 mM of HP-β-CD). Two previous works reported solubility increments of 147 times (at 37 °C) and 160 times (at 27 °C); however, they used at least 10 times greater HP-β-CD concentrations than us [30,31].

We did not surpass 10 mM of CD based on Do and coworkers’ data that reported aggregates starting at HP-β-CD concentrations higher than 12 mM [32], which could influence the complexation and Ks values [33]. For the 1:1 stoichiometry of complexation between HP-β-CD-CAP (as indicated by the AL type diagram), the calculated Ks between HP-β-CD and CAP is 2400 ± 221 M^−1^. Such Ks reveals a strong interaction between the CD and CAP [34], in accordance with the other mentioned works (1822 M^−1^ [31] and 966 M^−1^ [35]). The main goal of adding CDs to injectable products is to increase the drug water solubility, replacing the use of co-solvents [36]. However, it is important to use the minimum CD concentration necessary to achieve the solubilizing effect [20]. Thus, we determined the complexation efficiency (CE) from phase solubility diagrams (Equation (2), CE = 0.33), indicating that each complexed CD need an additional three “free” CD molecules to make one CAP molecule soluble (assuming the 1:1 stoichiometry of complexation). This CE value equals the average value observed by Loftsson et al. with dozens of drugs evaluated [20]. In accordance, drug:CD ratio in the formulation (D:CD, Equation (3)) indicates that each mole of CAP requires 4.2 moles of HP-β-CD to solubilize itself. The dynamic behavior of drug-CD complexes, maintained by weak van der Waals forces, justifies the need for excess CD to keep the complexed fraction [37]. Similar results were reported for other compounds such as terfenadine, miconazole, and clotrimazole, which demanded 4, 12, and 21 molecules of HP-β-CD, respectively, for optimal complexation [20]. Therefore, the aqueous solution of HP-β-CD-CAP was prepared in a molar ratio of 4.2 HP-β-CD to one CAP, subsequently freeze-dried, and characterized by solid state techniques.

### 3.2. Isothermal Titration Calorimetry

Isothermal titration calorimetry (ITC) and NMR support a deeper investigation of the association phenomenon and stoichiometry between CAP and HP-β-CD. ITC measures the heat generated or absorbed from the interaction between two molecules. Each addition of HP-β-CD on CAP solution led to complexation and consequent heat release (Figure 1B, upper panel). The exothermic reaction resulted from the interaction between CAP and HP-β-CD, with an enthalpy variation (ΔH) of −0.7 kcal/mol. The graph shows that most HP-β-CD molecules bond after the first injections because of CAP excess. As the concentration of free CAP molecules decreased, heat release diminished along the titration, indicating decrease of complex formation. 

Peak integration of ITC thermogram resulted in Figure 1B; the data was adjusted for the one-site model, which showed the best fit as expected by the phase solubility study. The affinity constant (*Kc*) of HP-β-CD-CAP determined by ITC was = 5100 M^−1^ with a stoichiometry of *n* = 1.1. Overall, ITC confirmed a 1:1 stoichiometry and a good binding complexation of HP-β-CD-CAP in solution [38]. Regarding the stability constant, ITC presented a two times higher value than phase solubility study. The phase solubility test is the most common one; however, it is performed under drug-saturated media, maintaining a high thermodynamic activity of the complex and allowing non-ideal solution effects [20]. The ITC analysis happens in the other extreme, with very diluted solutions, besides the frequent need of a co-solvent, such as in our experiment. Moreover, the dilution does not favor cyclodextrin aggregates or interactions between the complexes, as in phase solubility studies, whereas both interactions influences drug solubility [20].

### 3.3. Development of Freeze-Dried HP-β-CD-CAP

The major challenges in the pharmaceutical use of CAP include its low stability and water solubility [39]. The freeze-drying (or lyophilization) process can increase product stability by diminishing water available for chemical reactions or microbial growth [40]. Another advantage is the possibility of resuspending the complex in any local anesthetic solution, allowing flexibility and association without drug dilution. Therefore, we chose to lyophilize the HP-β-CD-CAP complex.

**Figure 2:**
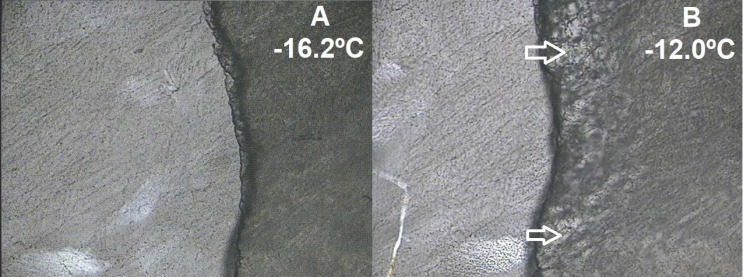
Selected frames during freeze-dry microscopy of the HP-β-CD-CAP complex (40× zoom). (**A**) Drying under temperature increments, frame at −16.2 °C. Brighter area = frozen matrix; darker area = dried matrix; dark dividing line = drying front. (**B**) Full collapse of the drying matrix.

An optimized lyophilization process ensures better product stability, shorter processing cycles, and lower costs [41]. Process enhancement relies on product physicochemical properties, such as the collapse temperature (Tc), determined by freeze-drying microscopy, and solid morphology, determined in SEM and PXRD experiments. To initiate drying above Tc generally results in structure collapse of the freeze-dried cake, which could impact residual moisture, reconstitution times, and storage stability [21]. The frozen structure collapsed at −12 °C (Figure 2B), similar to what was found for pure HP-β-CD in another study (15% m/w, −9.5 °C) [21]. The resultant Tc is also adequate for a further upscale, since most industrial freeze-dryers do not operate at temperatures below −40 °C.

### 3.4. Freeze-Dried CAP Complex

The freeze-dried CAP complex did not show loss/degradation of CAP, determined by HPLC. The resultant cake was white, porous, non-adherent, with rectilinear topology and uniform texture (no shrinkage or collapse). The cake was reconstituted with ultrapure water or mepivacaine solution in less than 10 s, an adequate time for parenteral freeze-dried presentations. The average residual moisture was 7.3% ± 0.9%; a similar result was found for freeze-dried tolbutamide/HP-β-CD (7.4%), while melphalan formulated with sulfobutylether-β-CD presented 4.5% moisture residue [42,43]. The product was also stable for at least a 12-month period, as described in Table 1. Although some papers mentioned freeze-drying of HP-β-CD-CAP for solid state studies, we believe this is the first work describing a process study that lead to a stable lyophilized complex product.

### 3.5. Characterization of the HP-β-CD-CAP Complex

#### 3.5.1. Differential Scanning Calorimetry

**Figure 3:**
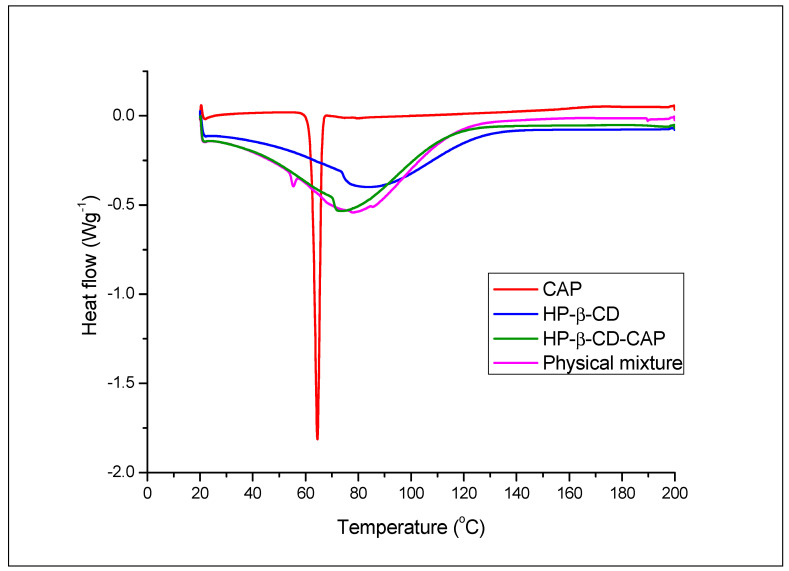
Differential scanning calorimetry (DSC) thermograms of capsaicin (CAP), HP-β-CD, HP-β-CD-CAP, and HP-β-CD-CAP physical mixture. Heating cycle from 20 to 200 °C, rate 5 °C/min, under inert atmosphere. The physical mixture contains the same amount of guest and host molecules of the complex, but they were not solubilized in water neither freeze-dried.

Comparison of the heating curves of pure compounds, CD complex, and physical mixture allows the identification of interactions between the guest and host molecules due to complex formation [44]. Hence, Figure 3 shows the heat curve of CAP, HP-β-CD, HP-β-CD-CAP, and physical mixture. CAP had a sharp endothermic event at 62.30 °C attributed to its melting point. HP-β-CD exhibited a broad endothermic event, with an onset at 73.75 °C, related to the loss of water molecules absorbed/bonded to the inner macrocyclic ring of CD [45].

As expected, the freeze-dried complex (HP-β-CD-CAP) showed no CAP melting peak, which is a result of the solid-state interaction. Only a broad endotherm event at 70.03 °C was observed, suggesting that all CAP molecules were complexed with CD. Contrarily, the physical mixture thermogram maintained the CAP and CD evidence, although both with a slight shift in temperature (CAP, 54.02 °C and HP-β-CD, 76.64 °C). The mixture thermogram confirmed that possible heating-induced interactions during DSC scan did not eliminate crystalline CAP. Considering the DSC results, it can be suggested total CAP amorphization is probably due to the formation of inclusion complexes. Further characterization (by PXRD and NMR) were performed to confirm this hypothesis.

#### 3.5.2. Powder X-ray Diffraction

**Figure 4:**
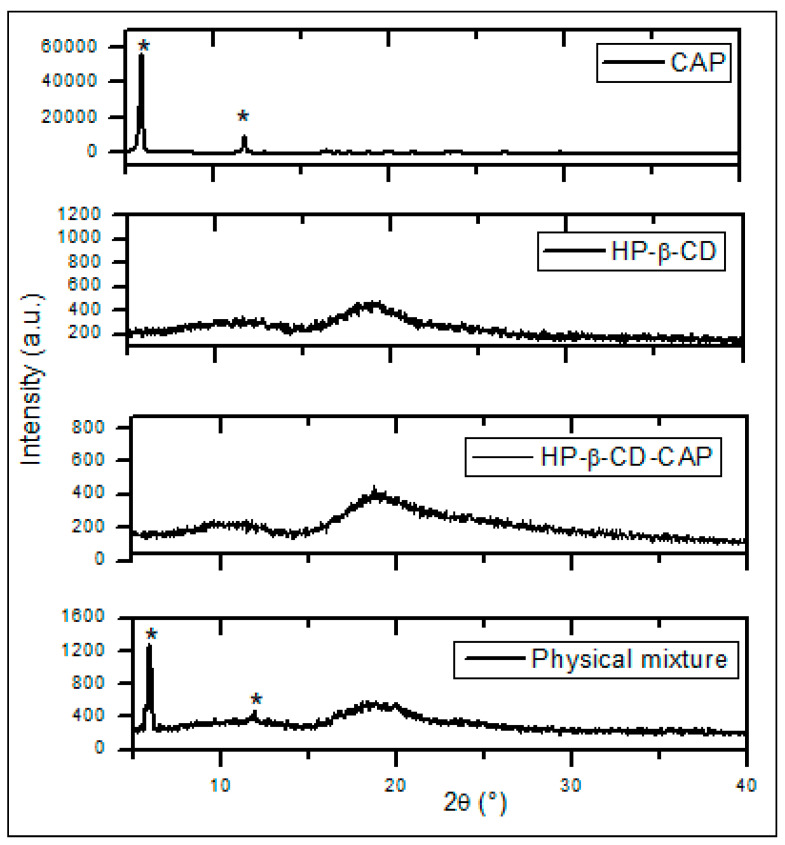
X-ray diffractograms of capsaicin (CAP), HP-β-CD, HP-β-CD-CAP, and physical mixture of HP-β-CD and CAP.

PXRD is widely used to characterize the crystallinity and degree of amorphization of CD complexes. Differences in the diffraction pattern of the free components, presumed inclusion complex, and physical mixture indicate interactions between components [44]. The diffraction patterns (Figure 4) confirmed the crystalline nature of CAP characterized by sharp and well-defined peaks (marked with asterisks), as showed in previous publications [31,35]. The HP-β-CD pattern indicates an amorphous structure, also reported before [46,47], whereas HP-β-CD-CAP freeze-dried diffractogram had no crystalline pattern, in contrast to the physical mixture.

The physical mixture reflected the sum of CAP crystalline pattern and the amorphous pattern of HP-β-CD (without complexation). Therefore, the sample with excess of CD (HP-β-CD-CAP) probably led to inclusion of all CAP molecules into HP-β-CD cavity, in agreement with DSC results.

#### 3.5.3. Scanning Electronic Microscopy

**Figure 5:**
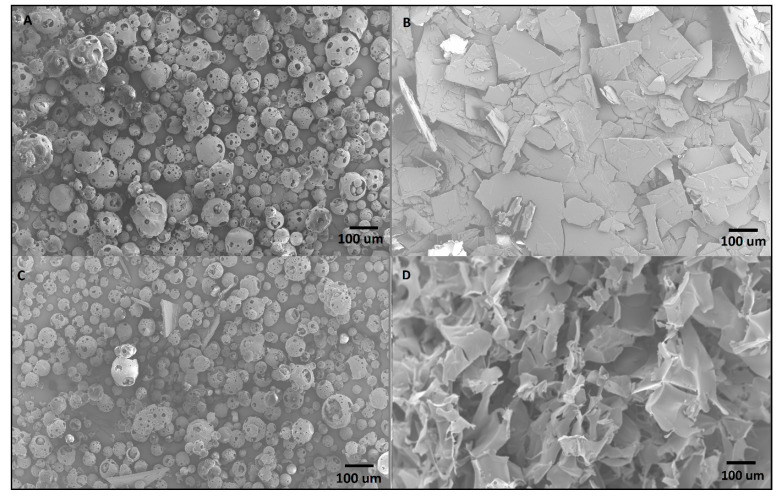
Scanning electron microscopy (SEM) micrographs of (**A**) HP-β-CD, (**B**) capsaicin (CAP), (**C**) physical mixture of HP-β-CD and CAP, (**D**) freeze-dried HP-β-CD-CAP.

The same samples from DSC and X-ray were then analyzed by SEM to evaluate their surface morphology. Figure 5A shows the HP-β-CD raw material displaying a spherical structure, with sizes in the range from 10 to 150 μm. Capsaicin raw material (Figure 5B) is organized as monoclinic crystals (rectangular plates), as expected [48]. The physical mixture in Figure 5C displayed fragments that resemble the individual components, confirming that simple mixture does not cause complexation or amorphization. Contrarily, the HP-β-CD-CAP complex (Figure 5D) image shows a homogeneous freeze-dried porous cake without no CAP crystals. These images support the X-ray and DSC data attesting amorphization of CAP by complexation with CD.

#### 3.5.4. Hydrogen Nuclear Magnetic Resonance

So far, ITC experiments have given thermodynamic information regarding the interaction of CAP and HP-β-CD. DSC, XRD and SEM provided evidence of the inclusion complex formation, in the solid state. However, NMR is the only technique that can directly prove the formation of the inclusion complex, either by the chemical displacement (^1^H-NMR) of the pure compounds or by ^1^H-^1^H proximities and other responses afforded specific pulse-sequences (e.g., NOE and DOSY).

**Figure 6:**
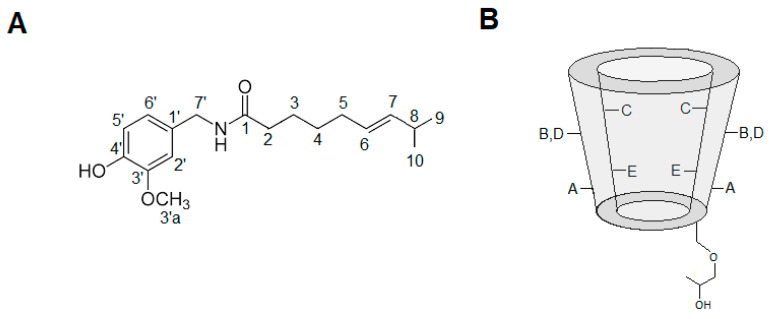
Representative structures and assignments of the hydrogen atoms of (**A**) CAP and (**B**) HP-β-CD.

Structures and assignment of the hydrogen atoms of the CAP and HP-β-CD are given in Figure 6. Table 2 shows the chemical shifts of CAP hydrogens, either in solution or complexed with HP-β-CD, highlighting significant changes observed in some hydrogen signals. The variation of chemical shifts higher than 0.05 ppm are considered significant [49]. The CAP signals H9 and H10 (methyl groups) displayed higher chemical shifts variation (Δ = −0.07 ppm), followed by the peaks H3 (Δ = −0.05 ppm), also in the CAP aliphatic chain. Hydrogens 2 and 8 showed variation 0.05 ppm, but their signals are partially overlapped (each other) at 2.19 ppm. In general, the aromatic hydrogens (2′, 5′, 6′) showed small displacements. Unfortunately, the signal of the methyl-ether hydrogens (3′a) got superposed with signals from HP-β-CD hydrogen E, restricting determination of changes in their chemical shift upon complexation. Therefore, the chemical displacements suggest insertion of hydrogens from the long hydrophobic chain of CAP (e.g., methyl hydrogens 9, 10) into the HP-β-CD cavity.

#### 3.5.5. Rotating Frame Overhauser Enhancement Spectroscopy (ROESY)

**Figure 7:**
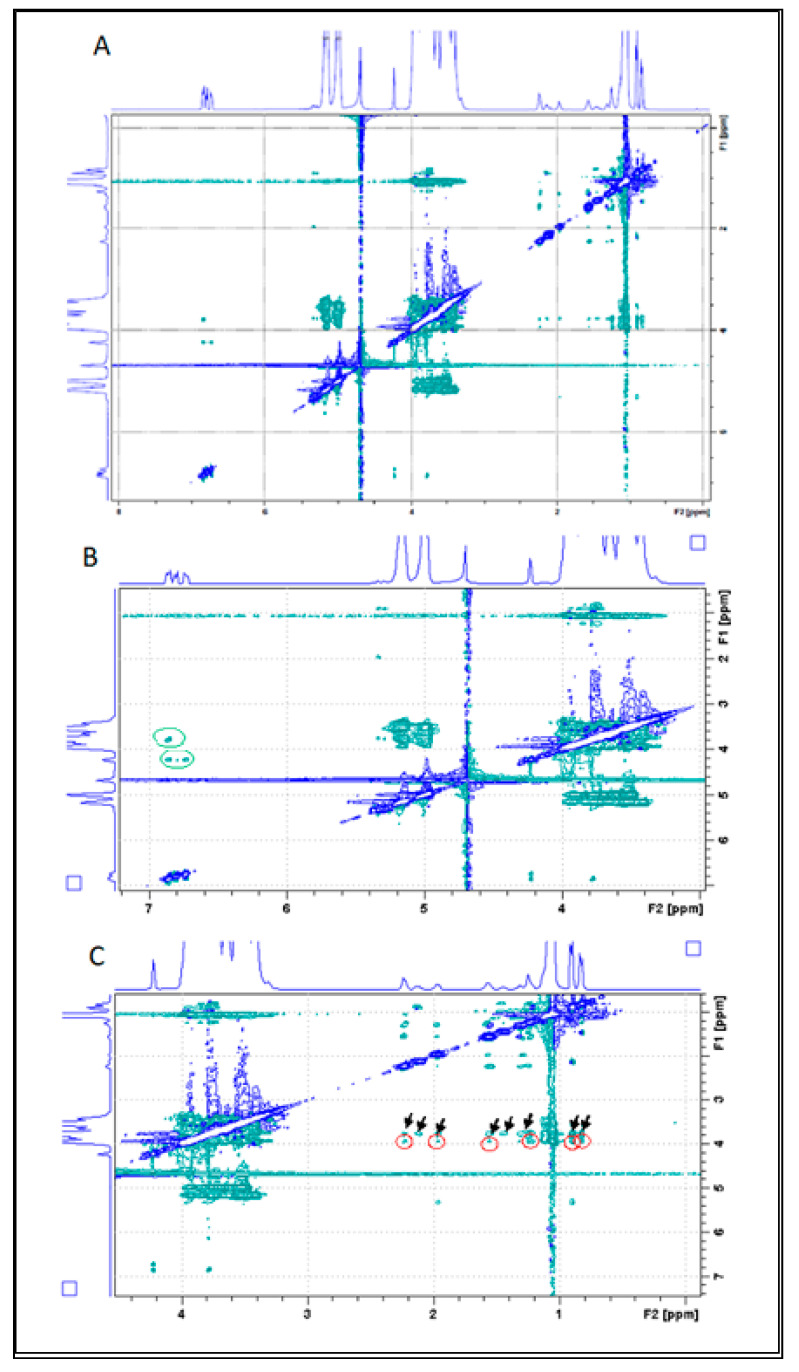
(**A**) 2D Rotating Frame Overhauser Enhancement Spectroscopy (ROESY) spectrum of the HP-β-CD-CAP complex (400 MHz, D_2_O/residual H_2_O reference was set to 4.7 ppm). (**B**) First spectrum expansion, in the region between 3 and 7 ppm. (**C**) Second spectrum expansion, in the region between 1 and 4 ppm.

To further elucidate the interaction between CAP and HP-β-CD, a ROESY-2D experiment was performed. The ROESY sequence allows detection of cross-peak correlations in the spectrum, representing hydrogens from the guest and host molecules that are close in space (less than six angstroms apart) but not covalently linked [49,50]. In the first expansion of the ROESY two-dimensional spectrum (Figure 7B) it is possible to observe the Nuclear Overhauser effect cross-peaks—green circles—that reveal spatial and non-scalar proximities between nuclei. The upper circle corresponds to the (intramolecular) interaction between CAP aromatics H2′, H5′, H6′ at 6.88 ppm and the H3a’ methyl-ether of CAP at 3.75 ppm. Additionally, the lower green circle corresponds to an intramolecular interaction between CAP aromatics H2′, H5′, H6′ at 6.88 ppm and H7′ of CAP (4.24 ppm).

Most interestingly, in the second expansion (Figure 7C) intermolecular interactions were detected (red circles) between H2 = 2.24 ppm, H5 = 1.97 ppm, H3 H = 1.57 ppm, H4 = 1.24 ppm, H9, 10 = 0.90 ppm of CAP and hydrogen C = 3.94 ppm of HP-β-CD. Hydrogen C is located in the inner HP-β-CD cavity macrocyclic ring and, along with hydrogen E, is very sensitive to changes in the chemical environment provoked by insertion of the guest molecules [49]. Additional cross-peaks (black arrows in Figure 7C) could be interpreted as a possible interaction between the same hydrogens of CAP (2, 5, 3, 4, 9, 10) and hydrogen E of HP-β-CD at 3.79 ppm. It is therefore very likely that upon complexation, the aliphatic portion of CAP gets inserted into the CD cavity. This ROESY results corroborated with the chemical shift variation present in the Table 2. The NMR results confirmed the inclusion complex formation between CAP and HP-β-CD and revealed details on the topology of the 1:1 stoichiometry complexation.

#### 3.5.6. Diffusion Ordered Spectroscopy—DOSY

As expected, the DOSY spectra of the free compounds revealed quite different diffusion coefficients (D) for HP-β-CD (2.00 × 10^−10^ m^2^s^−1^) and CAP (4.94 × 10^−10^ m^2^s^−1^). The slower diffusion of HP-β-CD reflects its larger structure when compared to free CAP. Similar diffusion coefficients for CAP (4.81 × 10^−10^ m^2^s^−1^) and HP-β-CD (2.21 × 10^−10^ m^2^s^−1^) were reported in the literature [51,52]. Small molecules such as CAP have a high diffusion coefficient, however, when complexed, their diffusion is retarded. This can be observed for the HP-β-CD-CAP complex, which diffusion coefficient value (2.35 × 10^−10^ m^2^s^−1^) is suggestive of the inclusion complex formation.

From the diffusion values it was possible to determine the CAP complexed fraction and the association constant, using Equations (5) and (6). The F_complexed_ of CAP bound to the complex was very high (F_complexed_ = 88%) and the *Ka* was 1079 M^−1^. Our group measured F_complexed_ and association constants for the complexation of different LA and CDs, using DOSY-NMR. For the complexation with HP-β-CD and the local anesthetic S-bupivacaine, the F_complexed_ = 57% and Ka = 91 M^−1^ [49]; and for oxethazaine, F_complexed_ = 38% and Ka = 198 M^-1^ [51]. Comparing to those LA, the HP-β-CD-CAP complex has a Ka and F_complexed_ notably higher. Together, all analytical techniques for characterization of HP-β-CD-CAP complex in aqueous solution revealed a strong intermolecular interaction. 

### 3.6. In Vitro Release Kinetics

**Figure 8:**
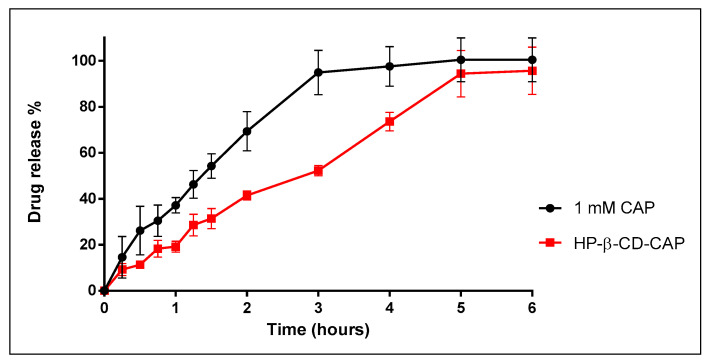
Cumulative release of capsaicin (CAP), free and complex with HP-β-CD, at 37 °C, *n* = 3.

Here, it was evaluated if the strong interaction in HP-β-CD-CAP complex could influence the release of the drug, hoping to better understand the following in vivo result. A freeze-dried complex only dissociates by heating or dilution/dissolution, which can displace the drug from CD cavity [53]. Therefore, the lyophilized cake (HP-β-CD-CAP) was resuspended in ultrapure water, the preferred solvent for further injection. According to Figure 8, the formulation containing HP-β-CD-CAP released CAP in a sustained manner when compared to CAP in solution (3 and 5 h, respectively).

In solution, as in Franz cells, the interaction of the drug with the CD cavity depends on weak non-covalent interactions, and therefore the complex continuously forms and dissociates [54]. However, when a formulation is injected into tissue, as in a local anesthetic infiltration, other factors affect the release of the drug, such as drug–protein binding, partition of the drug into the tissue, endogenous competitors for the CD site, elimination of CD, or effects related to pH or temperature [55]. Therefore, one cannot predict CAP release behavior in vivo by this in vitro test.

This work aimed to evaluate the anesthetic effect of mepivacaine when associated with freeze-dried HP-β-CD-CAP. Hence, the injection solution contained HP-β-CD-CAP (complex), excess HP-β-CD, and mepivacaine molecules that could interact with the excess CD or displace CAP from the complex. Dollo et al. reported *Ks* (from phase solubility study) values for LA (base form) system with HP-β-CD as: bupivacaine (95 M^−1^), lidocaine (19 M^−1^), mepivacaine (38 M^−1^) [56]. Therefore, affinity of HP-β-CD with LA is far smaller than with CAP. Additionally, the salt form of LA in aqueous solution contains most ionized drug which forms even less stable CD complexes than those described by Dollo 33. Consequently, it is expected that mepivacaine (MVC) molecules will not disturb HP-β-CD-CAP complex stability.

### 3.7. Evaluation of Analgesia in Inflamed Tissue

**Figure 9:**
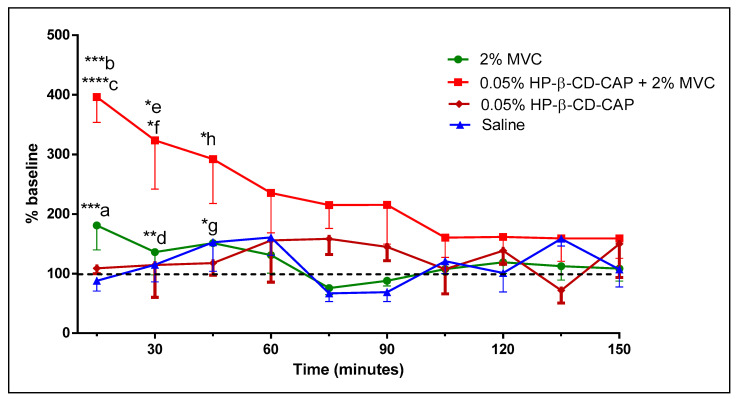
Measurements of mechanical sensitivity of mice after carrageenan-induced hyperalgesia. Basal behavior was tested after 4 h of carrageenan. Treatments include injection of 2% mepivacaine (MVC), 0.05% HP-β-CD/CAP + 2% MVC, 0.05% HP-β-CD-CAP and saline solution (placebo). Statistics: One-Way Anova/Tukey Test, * *p* < 0.05, ** *p* < 0.01, *** *p* < 0.0005 and **** *p* < 0.0001. a) 0.05% HP-β-CD-CAP + 2% MVC × 2% MVC; b) 0.05% HP-β-CD-CAP + 2% MVC × 0.05% HP-β-CD-CAP; c) 0.05% HP-β-CD-CAP + 2% MVC 2% × Saline; d) 0.05% HP-β-CD-CAP + 2% MVC × MVC 2%; e) 0.05% HP-β-CD-CAP + 2% MVC × 0.05% HP-β-CD-CAP; f) 0.05% HP-β-CD-CAP + 2% MVC × Saline; g) 0.05% HP-β-CD-CAP + 2% MVC × 2% MVC; h) 0.05% HP-β-CD-CAP + 2% MVC × 0.05% HP-β-CD-CAP.

The carrageenan-induced hyperalgesia model was used to evaluate the anesthetic action in the presence of acute inflammation. Pain threshold was measured against a mechanical stimulus on the inflamed plantar surface of a mice. We found that injection of 2% MVC solution did not induce anesthesia in mice at any time tested, like the placebo (Figure 9). As expected, the LA (MVC) failed to numb the inflamed tissue. However, the formulation containing 0.05% HP-β-CD-CAP + 2% MVC induced analgesia up to 45 min, despite the presence of inflammation. The results corroborated our hypothesis that TRPV1 channels activated by CAP would allow cell uptake of protonated mepivacaine, which is normally not permeable to the neuronal membrane [57].

## 4. Conclusions

To achieve enough local anesthesia in the presence of inflammation is still a challenge. Strategies such as buffering or addition of vasoconstrictors in the injection solution, pretreatment with anti-inflammatories, drug delivery system, and supplemental anesthesia (administration of other LA agents) did not satisfactorily increase the effect of LA in inflamed tissues in all situations [5]. Here, we described a novel combination of local anesthetics (mepivacaine) and CAP (complexed with HP-β-CD) that improved anesthesia when injected in inflamed tissue. In addition, we developed a reproducible, water soluble, stable and scalable formulation of CAP by its complexation with HP-β-CD, followed by freeze drying. This study shows the formulation as a promising alternative for future clinical assessments of local anesthetics and CAP. Nevertheless, further studies should be performed to address the toxicity of this drug delivery system as a parental formulation.

## Figures and Tables

**Table 1:** Stability study of HP-β-CD-CAP freeze-dried samples.

Specification	3 Months	6 Months	9 Months	12 Months
Cake appearance * (white, uniform, no collapse)	met the criteria	met the criteria	met the criteria	met the criteria
Reconstitution time ** (<10 s)	met the criteria	met the criteria	met the criteria	met the criteria
pH (5–8)	6.2 ± 0.1	6.4 ± 0.1	6.5 ± 0.1	6.1 ± 0.0
CAP content *** (95–105%)	99.2 ± 1.5	102.5 ± 0.6	103.2 ± 0.5	96.3 ± 3.4

* visual inspections, ** evaluated after addition of 4 mL of ultrapure water, *** HPLC determination, data representing mean ± SD (*n* = 3).

**Table 2:** 1H-NMR: Chemical shifts (in ppm) of peaks from CAP, in solution or complexed with HP-β-CD. See Figure 7 for assignment.

Capsaicin		HP-β-CD		HP-β-CD-CAP		Δ (ppm)
H atom	ppm	H atom	ppm	H atom	ppm	
9	0.83			9	0.90	−0.07
10	0.83			10	0.90	−0.07
4	1.24			4	1.24	0
3	1.52			3	1.57	−0.05
5	1.87			5	1.97	−0.1
2	2.19			2	2.24	−0.05
8	2.19			8	2.24	−0.05
		D	3.41		3.42	−0.01
		B	3.65		3.65	0
		E	3.77		-	-
3′a	3.78			3′a	-	-
		C	3.95		3.94	−0.01
7′	4.21			7′	4.24	−0.03
		A	5.18		5.17	−0.01
6	5.32			6	5.33	−0.01
7	5.32			7	5.33	−0.01
Aromatic (2′,5′,6′)	6.89–6.75			Aromatic (2′,5′,6′)	6.88–6.73	−0.01–0.02
